# Addressing NCDs: Protecting Health From Trade and Investment Law: Comment on "Addressing NCDs: Challenges From Industry Market Promotion and Interferences"

**DOI:** 10.15171/ijhpm.2019.41

**Published:** 2019-06-02

**Authors:** Krycia Cowling, Daniel Magraw

**Affiliations:** ^1^ Washington, DC, USA.; ^2^ Paul H. Nitze School of Advanced International Studies, Johns Hopkins University, Washington, DC, USA.

**Keywords:** Non-communicable Diseases, Trade, Investment, International Law, Health Policy

## Abstract

Building on Tangcharoensathien and colleagues’ description of four tactics used by the tobacco, alcohol, processed food, and breast milk substitute industries to interfere with the development and implementation of health policies, we present a fifth tactic: trade and investment disputes. We describe recent examples of trade and investment claims filed by the tobacco industry to challenge plain packaging legislation, which may serve as a model for future claims by this and other industries. Next, we clarify specific areas of potential conflict between non-communicable disease (NCD) control policies and trade and investment agreement (TIA) commitments, identifying possible vulnerabilities that may be exploited by industry to challenge the legality of these policies. We conclude with ideas to strengthen the position of health policies vis-à-vis commitments in TIAs.

As described by Tangcharoensathien and colleagues in their editorial “Addressing NCDs: Challenges from Industry Market Promotion and Interferences,” tactics deployed by the tobacco industry to interfere with the development and implementation of legitimate health policies are increasingly also utilized by the alcohol, processed food, and breast milk substitute industries.^1^ If these industries continue to follow the trajectory of the tobacco industry, they can be expected to utilize trade and investment disputes as an additional avenue of potential attack, by exploiting commitments in trade and investment agreements (TIAs) in attempts to undermine health policies. In addition to the four types of industry tactics described by Tangcharoensathien and colleagues, challenges from claims under TIAs comprise a fifth category the public health community should be alerted to as it works to develop policies to reduce consumption of tobacco, alcohol, highly processed foods, and breast milk substitutes when breast milk is available.

## Recent Examples From the Tobacco Industry

In the case of tobacco, trade and investment disputes have recently occurred through two mechanisms. Corporations have directly sued governments through what is known as investor-state dispute settlement, a provision included in most international investment agreements since the 1990s.^2^ A prominent example of this is *Philip Morris v. Uruguay*, in which the tobacco company Phillip Morris Products (Switzerland) filed a claim against Uruguay, arguing that its law requiring 80% of tobacco packaging be covered with a health warning label violated the company’s intellectual property rights under the Switzerland-Uruguay bilateral investment treaty.^3^ Second, industries have utilized their influence to encourage governments to file claims against other governments on behalf of their interests. This is the more long-standing mode of complaint, enshrined in the World Trade Organization (WTO) rules and provided for by the dispute settlement clauses of most trade agreements. A recent example is *Australia – Tobacco Plain Packaging (Honduras),* filed at the WTO by Honduras and several other tobacco-exporting countries over Australia’s plain packaging law for tobacco products, raising similar arguments as the investment case against Uruguay.^4^ The rulings in these two examples were ultimately in favor of the plain packaging laws, but were decided after legal battles costing the respondent countries millions of dollars and spanning several years. Those costs were not fully recovered: for example, only the costs of the arbitration process and about 70% of legal costs were ordered to be reimbursed by Phillip Morris in the investment case, leaving Uruguay to pay approximately US$3 million.^5^

It may come as a surprise to many in the public health community that the legality of such health policies is even questionable under international trade and investment rules. However, many of the “best buy” interventions endorsed by the World Health Organization (WHO) for combatting key non-communicable disease (NCD) risk factors^6^ arguably have the potential to be interpreted as interfering with TIA commitments. With almost 300 trade agreements^7^ and over 2000 investment agreements in force,^8^ and because cases decided under one agreement are not binding on subsequent cases (particularly those brought under other agreements), the potential for harassment is huge. As governments continue to consider and develop new policies to restrict advertising, enact product-specific taxes, mandate various types of health warning labels, and take other health-promoting measures, the possibility of challenges to these policies from the tobacco, alcohol, processed food, and breast milk substitute industries through the mechanisms created by TIAs should not be dismissed.

## Sources of Potential Conflict Between Health Policies and Trade and Investment Agreements

The key commitment in all TIAs is that foreign and domestic products and capital be accorded equal opportunity to participate in the national market, referred to as “non-discrimination.”^9^ A policy that differentially and disadvantageously affects the sale of a foreign good, service, or investment may be accused of being disguised protectionism, and raised as a possible violation of a trade or investment agreement commitment. This is a key potential point of contention for any measure that seeks to discourage consumption of a good or service based on its value for health – if, for example, items whose consumption are discouraged are disproportionately of foreign origin or produced with foreign capital, the policy can appear discriminatory. In many low- and middle-income countries, where sales of these products are increasing most rapidly, the majority of alcohol, tobacco, highly processed food, and breast milk substitute products are imported or domestically manufactured by foreign corporations,^10-12^ thereby creating the potential for policies intended to deter their consumption to appear discriminatory against imports or foreign investments. Ecuador provides a hypothetical example; the country passed a law in 2014 requiring “traffic light” nutrition labels on all packaged processed foods to inform consumers of each item’s sugar, salt, and fat content.^13^ However, these labels do not apply to prepared foods, such as fried plantains or pastries, sold in street kiosks or corner stores, which can also be high in salt, sugar, or fat. A manufacturer of processed foods could conceivably claim it is discriminatory to require nutrition labels for their products but not these unpackaged alternatives, whereby the policy favors locally made items. While this particular claim has not been made to date, such a line of argument can form the genesis of a trade or investment dispute.

In addition, TIAs typically contain norms disciplining member countries’ regulations that set health, environmental, and safety standards, even if these are non-discriminatory, and these norms have given rise to many challenges. For example, the WTO Agreement on Sanitary and Phytosanitary Standards provides that health standards should be no more trade restrictive than necessary; and the WTO Agreement on Technical Barriers to Trade, which governs packaging such as health-related labels, contains similar restrictions. Investment agreements typically require that laws, including those that regulate health, must be “fair and equitable,” a standard open to wide interpretation. Similarly, investment agreements typically require compensation for expropriations, including indirect ones. The standard for whether a regulation constitutes an expropriation – or seizure by the state – is also somewhat unclear. Health, safety and environmental regulations have been challenged on both “fair and equitable” and “expropriation” grounds, sometimes successfully.^4^ Thus, trade and investment rules such as these can narrow the permissible scope, and give rise to disputes about the validity, of health, safety, and environmental regulations, in addition to any concerns arising from whether these measures are perceived as discriminatory.

## The Evolving Non-communicable Disease Policy Landscape

The global landscape of NCD control policies is contentious, as commercial interests are threatened by new efforts to reduce sales and consumption of products that increase the risk of NCDs. Latin American countries have led the way in implementing front-of-pack nutrition labels,^14^ but records from meetings of the WTO Agreement on Technical Barriers to Trade committee indicate members have recently raised concerns about these policies in Ecuador^15^ and Peru,^16^ among others. Concerns have also been raised about a proposed policy in Thailand to limit the use of celebrities and cartoons on alcohol labels^17^ and a measure in Nepal to require graphic warnings or statements on alcohol beverages.^18^ While committee meetings are intended as a forum to resolve differences before reaching the level of a formal claim, these actions indicate that new NCD policies are closely monitored by industry and that countries are likely to face pressure to repeal existing policies or to weaken or not enact policies under consideration.

Other examples indicate that the risk is not only of claims being raised against health policies, but also the inclusion of ever-tighter requirements in new TIAs that further limit the scope of allowable measures. In the renegotiation of the North American Free Trade Agreement, it has been reported that the United States pressed to include language restricting the ability of member countries to enact measures that would require warning labels about health risks on highly processed “junk” foods and sugary beverages.^19^ On the other hand, some countries have taken the opportunity presented by the negotiation of new agreements to preempt industry challenges to health policies. For example, the recently signed Comprehensive and Progressive Agreement for Trans-Pacific Partnership bars claims by tobacco companies under the agreement.^20^

## Possible Ways Forward for Health Policy

Somewhat ironically, partial solutions, as well as early indications of the problem, may come from following the example of tobacco. The Framework Convention on Tobacco Control (FCTC), adopted in 2003 and considered “the first global public health treaty,” is an international agreement that commits countries Party to it to enact various measures to discourage tobacco use.^21^ While the FCTC has been criticized for not having stronger language on trade,^22^ it nevertheless provided important validation for Uruguay in its investment case by supporting its plain packaging measure with an international treaty commitment. The enactment of similar treaties to govern other high-risk products may likewise help to provide legal foundation to combat any relevant TIA claims. International agreements on alcohol^23^ and the marketing of junk food to children^24^ have been proposed. However, such agreements are not likely to be created easily given the powerful reach of the affected industries, as evidenced by the US’s recent opposition to a WHO resolution to limit inaccurate or misleading marketing of breast milk substitutes.^25^ However, inter-governmental organizations can play constructive roles even in the absence of new treaties. Uruguay’s case, for example, was buttressed by a report on labeling from the WHO.

In time, the severity of these concerns will become clear. In tobacco plain packaging disputes to date, dispute settlement panels have appropriately weighed health concerns, though the cost of defending those concerns has been unconscionably high. Hopefully, policies to combat the growing global NCD epidemic will continue to take precedence over claims of discrimination, loss of intellectual property rights, or other alleged violations of TIA commitments. Regardless of whether additional health measures are attacked via formal trade or investment claims, there is clear evidence that industry influence is already affecting the language of new treaties and pressuring countries as new health policies are being drafted. The public health community would be wise to take the offensive, by increasing awareness of this potential threat to NCD control; improving interdisciplinary public health education with insights from international law; and collaborating with trade and investment ministries to carefully design new health measures that are not trade restrictive and to bar these industries from bringing challenges under TIAs or requiring them to pay the full cost of defending against such claims. Pursuing additional product-specific treaties akin to the FCTC should be a priority to further support the international legal foundation for policies discouraging consumption of alcohol, highly processed foods, particularly sugar sweetened beverages, and breast milk substitutes in place of breast milk. Anticipating and blocking avenues of potential attack from industry, such as those posed by TIAs, are essential to an effective and sustainable global strategy to reduce NCDs.

## Ethical issues

Not applicable.

## Competing interests

The authors declare that they have no competing interests.

## Authors’ contributions

KC conceptualized and wrote the first draft of the manuscript. DM contributed to the manuscript and made critical revisions for important intellectual content.

## Authors’ affiliations

^1^Washington, DC, USA. ^2^Paul H. Nitze School of Advanced International Studies, Johns Hopkins University, Washington, DC, USA.
